# Metabolic fingerprinting of gilthead seabream (*Sparus aurata*) liver to track interactions between dietary factors and seasonal temperature variations

**DOI:** 10.7717/peerj.527

**Published:** 2014-08-26

**Authors:** Tomé S. Silva, Ana M.R. da Costa, Luís E.C. Conceição, Jorge P. Dias, Pedro M.L. Rodrigues, Nadège Richard

**Affiliations:** 1 SPAROS Lda., Olhão, Portugal; 2 CCMAR, Centre of Marine Sciences of Algarve, University of Algarve, Campus de Gambelas, Faro, Portugal; 3 CIQA, Algarve Chemistry Research Centre, University of Algarve, Campus de Gambelas, Faro, Portugal; 4 Department of Chemistry and Pharmacy, University of Algarve, Campus de Gambelas, Faro, Portugal

**Keywords:** Aquaculture, Gilthead seabream, Liver, Metabolomics, Winter disease, Winter syndrome, Thermal stress, Metabolic fingerprinting, Vibrational spectroscopy, FT-IR

## Abstract

Farmed gilthead seabream is sometimes affected by a metabolic syndrome, known as the “winter disease”, which has a significant economic impact in the Mediterranean region. It is caused, among other factors, by the thermal variations that occur during colder months and there are signs that an improved nutritional status can mitigate the effects of this thermal stress. For this reason, a trial was undertaken where we assessed the effect of two different diets on gilthead seabream physiology and nutritional state, through metabolic fingerprinting of hepatic tissue. For this trial, four groups of 25 adult gilthead seabream were reared for 8 months, being fed either with a control diet (CTRL, low-cost commercial formulation) or with a diet called “Winter Feed” (WF, high-cost improved formulation). Fish were sampled at two time-points (at the end of winter and at the end of spring), with liver tissue being taken for FT-IR spectroscopy. Results have shown that seasonal temperature variations constitute a metabolic challenge for gilthead seabream, with hepatic carbohydrate stores being consumed over the course of the inter-sampling period. Regarding the WF diet, results point towards a positive effect in terms of performance and improved nutritional status. This diet seems to have a mitigating effect on the deleterious impact of thermal shifts, confirming the hypothesis that nutritional factors can affect the capacity of gilthead seabream to cope with seasonal thermal variations and possibly contribute to prevent the onset of “winter disease”.

## Introduction

Metabolomics is usually defined as the holistic study of metabolites in living systems, which includes most molecules present apart from polynucleotides (mostly studied by genomics and transcriptomics) and proteins (mostly studied by proteomics). In a way, this field can be seen as an extension of analytical chemistry and chemometrics, given that the simultaneous study of the wide range of molecules present in cells and biological fluids only became possible with the continuous instrumentation and methodological developments in these areas. It is not surprising, then, that most metabolomic studies apply popular analytical chemistry techniques (from mass spectrometry and nuclear magnetic resonance, to optical spectroscopy, atomic spectroscopy and gravimetry, among others) optionally coupled to separation techniques (usually chromatography or electrophoresis-based) (Dunn & Ellis, 2005; Goodacre et al., 2004).

Metabolomic studies are often of a comparative nature and usually take either a metabolic profiling and/or a metabolic fingerprinting approach. In the first case, the focus is on obtaining a quantitative estimate of all (or a specific subset of the) metabolites present in a certain biological sample. Given the high complexity of biological samples, metabolic profiling usually requires high-resolution separation methods prior to analysis, which explains the popularity of “hyphenated mass spectrometry” methods (such as GC-MS, HPLC-MS and CE-MS) for this purpose (Dettmer, Aronov & Hammock, 2007; Scalbert et al., 2009). The second approach, metabolic fingerprinting, avoids altogether the deconvolution of the mixture into its different components and simply attempts to capture a multivariate fingerprint of a biological sample in some arbitrary feature space in a way that the similarity of samples in the “real” metabolomic feature space can be deduced/estimated from their similarity in the fingerprinting space. For this purpose, techniques such as ^1^H-NMR, ^13^C-NMR, DIMS (Direct Injection Mass Spectrometry), vibrational (FT-IR or Raman) spectroscopy and pyrolysis mass spectrometry have been commonly used, which, although not nearly as sensitive and specific as hyphenated methods, are often less expensive, quicker and/or require less sample preparation (Dettmer, Aronov & Hammock, 2007; Dunn & Ellis, 2005; Ellis et al., 2007).

This manuscript describes the results of a trial in which we explored the use of transmissive FT-IR spectroscopy for metabolic fingerprinting of liver tissue from gilthead seabream (*Sparus aurata*), to better understand how seasonal temperature variations and dietary factors affect the hepatic metabolic content. FT-IR spectroscopy (as other types of IR spectroscopy) is based on the differential absorption of IR radiation with specific wavelengths by different molecules. The characteristic wavelengths absorbed by a specific molecule depend on the energy differences between vibrational states of the molecule, being mostly defined by the presence of particular functional groups, as each functional group tends to display specific vibration modes, thereby conditioning the energy of allowed vibrational transitions (Ellis & Goodacre, 2006). Though the use of FT-IR spectroscopy as a metabolic fingerprinting technology in fish biology and aquaculture research is still incipient, several studies have already pointed out its usefulness in such diverse contexts as the differentiation between wild *vs.* farmed gilthead seabream (Ceylan, Tanrikul & Özgener, 2014), the study of the effects of estrogens and other pollutants in rainbow trout (Çakmak, Togan & Severcan, 2006; Çakmak et al., 2003), analysis of the effects of zinc and arsenic in indian carp (Palaniappan, Nishanth & Renju, 2010; Palaniappan & Vijayasundaram, 2008; Palaniappan & Vijayasundaram, 2009) and analysis of lipids in frozen hake fillets (Sánchez-Alonso, Carmona & Careche, 2012), underlying the flexibility of this technique in the study of distinct fish tissues within the context of several different biological problems.

As mentioned in the previous paragraph, the target of this study was the gilthead seabream, a species of high commercial value reared in the Mediterranean coast. The reason we are interested in studying the effect of seasonal variations in temperature on gilthead seabream metabolism is the occasional occurrence of a syndrome (called the “winter disease” or the “winter syndrome”), which induces chronic mortalities during the low temperature months, followed by acute mortality when the temperature progressively rises, with significant economic impact for fish farmers. It is characterized by both behavioral (e.g., erratic swimming, voluntary fasting, hyposensitivity to stimuli) and physiological (e.g., impaired growth, pale and fatty liver, tissue necrosis, infections) changes that can ultimately lead to systemic dysfunction and death (Contessi et al., 2006; Doimi, 1996; Gallardo et al., 2003; Ibarz et al., 2010b; Ibarz et al., 2010c; Kyprianou et al., 2010; Tort et al., 1998; Tort et al., 2004). In etiological terms, though many factors seem to be relevant to the onset of this syndrome (namely, the occurrence of metabolic distress, nutritional imbalances and/or deficits, immunosuppression and the presence of opportunistic pathogens (Contessi et al., 2006)), the common underlying factor seems to be exposure to low environmental temperatures, which induce slower metabolic rates and disrupts feeding behaviour (with fish displaying little to no feed consumption when temperatures drop below 13 °C).

Although the exposure of reared gilthead seabream to low temperatures might be unavoidable during cold months, there are indications that the administration of specifically-formulated diets prior, during and/or after low temperature periods might mitigate the ultimate impact of thermal challenge on fish nutritional status and health, helping to prevent the occurrence of full-blown “winter syndrome” (Bavčević et al., 2006; Ibarz et al., 2010a).

Within this context, a nutrient-enriched diet (“Winter Feed” or WF) was designed to serve as reference for a high-quality feed, appropriate to maintain improved nutritional and metabolic status in gilthead seabream during the cold months. Although hardly economically feasible, this diet was formulated to constitute a high-quality reference (against which other possible diets can be compared), containing a higher proportion of marine-derived ingredients (i.e., fish meal and krill protein hydrolysate) and supplemented with phagostimulants (i.e., betaine), marine phospholipids, soy lecithin, antioxidant vitamins and taurine, compared to a challenging diet with low levels of fishmeal and a concomitant partial replacement of fish oil by rapeseed oil (“CTRL” diet), representing a low-cost commercial formulation. The used formulations were thus chosen to induce (as far as possible) two extreme nutritional states in gilthead seabream so that we can confirm, on one hand, the possibility of modulating the challenging effect of seasonal temperature variations through a nutritional approach and, on the other hand, the feasibility of using FT-IR spectroscopy for the purposes of metabolic fingerprinting.

## Materials & Methods

### Experimental diets

A control feed (CTRL) was formulated with low-fishmeal levels (15%), a significant amount of plant-protein sources and a blend of fish and rapeseed oils (Table 1). This diet was supplemented with an inorganic phosphorus source (dicalcium phosphate) and a crystalline essential amino acid (L-lysine) to guarantee that known nutritional requirements of the species were covered. The CTRL diet contained 48.3% crude protein, 19.6% crude fat, 22.8 kJ/g gross energy. Comparatively, the experimental winter feed (WF) had a much higher proportion of marine-derived protein sources (45.8%), consequently lower level of plant-proteins and the totality of the oil fraction associated to fish oil and krill phospholipids. This WF diet was further supplemented with betaine as a phagostimulant (Kasumyan & Døving, 2003; Kolkovski, Arieli & Tandler, 1997), soy lecithin to facilitate fat emulsification during digestion and/or improve lipid clearance from the gut (Koven et al., 1993; Koven et al., 1998; Tocher et al., 2008), vitamin C and vitamin E as antioxidants and non-essential amino acid taurine, given its role as antioxidant and involvement on bile acid conjugation (El-Sayed, 2013). The WF diet contained 50.6% crude protein, 19.7% crude fat, 22.4 kJ/g gross energy.

**Table 1:** Ingredients and proximate composition of the experimental diets. Information on the formulation of the experimental diets in terms of ingredients, as well as post-extrusion instrumental estimates of nutrient composition.

	CTRL	WF
**Ingredients (% w/w)**		
Fishmeal 70 LT^a^	10	30
Fishmeal 60^b^	5	10.8
Krill protein hydrolysate^c^	0	5
Soy protein concentrate^d^	8	0
Pea protein concentrate^e^	4	0
Corn gluten^f^	16	5.5
Wheat gluten^g^	8.4	0
Soybean meal 48^h^	16.5	7
Wheat meal	5	12.5
Rapeseed meal	4	0
Aquatex G2000 (bran)^i^	2	3
Fish oil^j^	10	8
Rapeseed oil^j^	5.7	0
Krill PPC^k^	0	12.5
Soy lecithin^l^	0	1
Guar gum (binder)	0.5	0.5
Vit & Min Premix PVO 40/02	0.2^m^	0.3^n^
DCP^o^	4	1
Lutavit C35^p^	0	0.3
Lutavit E50^q^	0.1	0.5
L-Lysine^r^	0.5	0
L-Taurine^s^	0	1
Choline chloride	0.1	0.1
Betaine^t^	0	1
**Proximate composition**		
Dry matter (DM), %	97.5	94.3
Crude protein, % DM	48.3	50.6
Crude fat, % DM	19.6	19.7
Ash, % DM	8.2	10.9
Gross Energy, MJ/kg	22.8	22.4
Phosphorus, % DM	1.5	1.7

**Notes.**

a Peruvian fishmeal LT: 670 g kg^−1^ crude protein (CP), 90 g kg^−1^ crude fat (CF), EXALMAR, Peru.

b Fish by-products meal: 540 g kg^−1^ CP, 80 g kg^−1^ CF, COFACO, Portugal.

c Krill protein hydrolysate: >700 g kg^−1^ CP, <30 g kg^−1^ CF, OLYMPIC SEAFOOD AS, Norway.

d Soycomil PC: 630 g kg^−1^ CP, <10 g kg^−1^ CF, ADM, The Netherlands.

e Lysamine GP: 780 g kg^−1^ CP, 80 g kg^−1^ CF, ROQUETTE, France.

f GLUTALYS: 610 g kg^−1^ CP, 80 g kg^−1^ CF, ROQUETTE, France.

g VITEN: 857 g kg^−1^ CP, 13 g kg^−1^ CF, ROQUETTE, France.

h Solvent extracted dehulled soybean meal: 470 g kg^−1^ CP, 26 g kg^−1^ CF, SORGAL SA, Portugal.

i Dehulled grinded pea grits: 240 g kg^−1^ CP, <10 g kg^−1^ CF, SOTEXPRO, France.

j Henry Lamotte Oils GmbH, Germany.

k Krill PPC (25–30% phospholipids): 450 g kg^−1^ CP, 500 g kg^−1^ CF, OLYMPIC SEAFOOD AS, Norway.

l Yelkinol AC (65% phospholipids): 750 g kg^−1^ CF, ADM, The Netherlands.

m Premix for marine fish, PREMIX Lda, Portugal. Vitamins (IU or mg/kg diet): sodium menadione bisulphate, 10 mg; retinyl acetate, 8000 IU; DL-cholecalciferol, 1700 IU; thiamin, 8 mg; riboflavin, 20 mg; pyridoxine, 10 mg; cyanocobalamin, 0.02 mg; nicotinic acid, 30 mg; folic acid, 6 mg; inositol, 300 mg; biotin, 0.7 mg; calcium panthotenate, 70 mg; betaine, 400 mg. Minerals (mg/kg diet): cobalt carbonate, 0.1 mg; copper sulphate, 5 mg; ferric sulphate, 60 mg; potassium iodide, 1.5 mg; manganese oxide, 20 mg; sodium selenite, 0.25 mg; zinc oxide, 30 mg; sodium chloride, 80 mg; excipient: wheat middlings.

n Premix for marine fish, PREMIX Lda, Portugal. Vitamins (IU or mg/kg diet): sodium menadione bisulphate, 15 mg; retinyl acetate, 12000 IU; DL-cholecalciferol, 2250 IU; thiamin, 12 mg; riboflavin, 30 mg; pyridoxine, 15 mg; cyanocobalamin, 0.03 mg; nicotinic acid, 45 mg; folic acid, 9 mg; inositol, 450 mg; biotin, 1.05 mg; calcium panthotenate, 105 mg; betaine, 600 mg. Minerals (mg/kg diet): cobalt carbonate, 0.15 mg; copper sulphate, 7.5 mg; ferric sulphate, 90 mg; potassium iodide, 2.25 mg; manganese oxide, 30 mg; sodium selenite, 0.38 mg; zinc oxide, 45 mg; sodium chloride, 120 mg; excipient: wheat middlings.

o Dicalcium phosphate: 18% phosphorus, 23% calcium, Fosfitalia, Italy.

p Vitamin C: >35% sodium and calcium salts of ascorbyl-2-phosphate, BASF, Germany.

q Vitamin E: >50% DL-alpha-tocopheryl acetate, BASF, Germany.

r L-Lysine HCl 99%: Ajinomoto Eurolysine SAS, France.

s L-Taurine 99%: Ajinomoto Eurolysine SAS, France.

t Betafin S1 (>96% betaine): DANISCO, Denmark.

Main ingredients were ground (below 250 micron) in a micropulverizer hammer mill (Hosokawa Micron, SH1, The Netherlands). Powder ingredients and oil sources were then mixed according to the target formulation in a paddle mixer (Mainca RM90, Spain). Diets were manufactured by temperature controlled-extrusion (pellet size: 5.0 mm) using a low shear extruder (Italplast P55, Italy). Upon extrusion, all feed batches were dried in a convection oven (OP 750-UF; LTE Scientifics, United Kingdom) for 2 h at 60 °C. Throughout the duration of the trial, experimental feeds were stored at room temperature, but in a cool and aerated emplacement. Samples of each diet were taken for analysis of proximate composition (Table 1).

### Fish rearing and sampling

**Figure 1:**
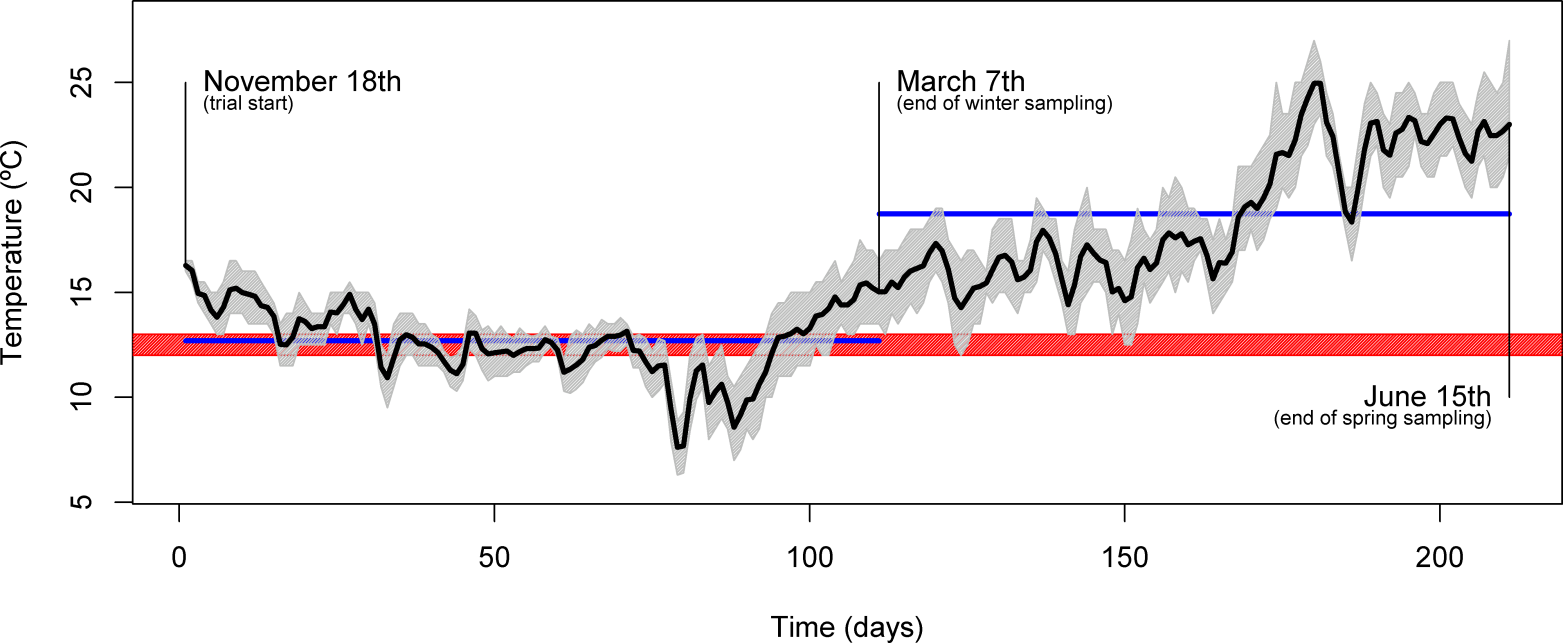
Seasonal temperature profile. Plot showing the daily mean water temperature (black line) throughout the trial. The full range of temperatures are denoted by the area shaded in gray. Relevant dates (trial start, 1st sampling and 2nd sampling) are shown directly in the plot. The blue horizontal lines indicate the mean temperature over the course of the two inter-sampling periods. The red shading indicates the temperature threshold below which gilthead seabream generally display voluntary fasting (12–13 °C).

The experiment was conducted at the Experimental Research Station of CCMAR (37°00′ N, 07°58′ W, Faro, Portugal) and took place between November and June of the following year. Four homogenous groups of 25 gilthead seabream (*Sparus aurata*) each, with a mean initial body weight of 87 ± 5 g, were stocked in 1000 L outdoor circular plastic tanks supplied with flow-through seawater (rearing density of about 2.18 kg m^−3^). Throughout the trial, fish were subjected to a natural temperature regime, which was logged every hour (Fig. 1), with mean daily temperatures ranging from 7.6 °C to 25.0 °C. Similarly, other physicochemical parameters varied within the natural ranges (natural photoperiod, salinity: 33 ± 2%, dissolved oxygen: above 5 mg L^−1^). Each dietary treatment was tested in duplicate tanks over 213 days. Fish were fed to apparent satiety, by hand, either once a day (at 10.00 h, during the winter period), or twice a day (at 10.00 and 16.00 h, during the spring period) and feed intake was recorded. Prior to harvesting for sampling, fish were starved for 48 h.

Over its course, two samplings were performed: in March (end of winter sampling), during the lowest temperature regime, and in June (end of spring sampling), during the temperature rise period (Fig. 1). Fish were weighted, measured and liver samples (from 10 fish/tank) taken and immediately frozen in liquid nitrogen for further characterization. At sampling, macroscopic observation of liver allowed identification of abnormal characteristics (discoloration, firmness and exudation). Analysis of whole-body composition was performed on the fish carcasses after liver excision.

The experiment described was conducted in accordance with the Guidelines of the European Union Council (Directive 2010/63/EU) and the Portuguese legislation for the use of laboratory animals, and under a “Group-1” licence (permit number 0420/000/000-n.99-09/11/2009) from the Veterinary Medicine Directorate, the competent Portuguese authority for the protection of animals, Ministry of Agriculture, Rural Development and Fisheries, Portugal.

### Proximate composition of feeds and whole fish

Proximate composition analysis of the diets and whole fish was performed by the following methods: dry matter, by drying at 105 °C for 24 h; ash, by combustion at 550 °C for 12 h; crude protein (N × 6.25), by a flash combustion technique followed by gas chromatographic separation and thermal conductivity detection (LECO FP428); fat, after petroleum ether extraction, by the Soxhlet method; total phosphorus, according to the ISO/DIS 6491 method, using the vanado-molybdate reagent; gross energy, in an adiabatic bomb calorimeter (IKA).

### Solid-phase transmissive FT-IR spectroscopy

Liver tissue samples from 10 fish per tank (i.e., 20 fish per dietary treatment), which were collected at each of the two distinct sampling times (March and June), were lyophilised, ground in liquid nitrogen and lyophilised again, reducing them to a fine dry powder. Using an agate pestle and mortar, each liver sample was then mixed with KBr (following a ratio of 500 mg KBr per 5 mg sample) until homogeneous. Small amounts of these mixtures were then placed in an evacuated die (13 mm diameter) and subjected to a pressure of about 6 × 10^6^ Pa for 8 min, in order to obtain clear 1 mm-thick pellets for further analysis by transmissive IR spectroscopy.

Four spectra per pellet were acquired (at distinct points of the pellet) using a “TENSOR” FT-IR equipment (Bruker) coupled to the OPUS control/analysis software (Bruker). Each of these spectra was obtained by averaging 25 spectra covering the 400–4000 cm^−1^ range, at a resolution of 4 cm^−1^. Spectra were then post-processed by the application of a baseline subtraction algorithm (rubberband correction, 64 points) and exported from OPUS for statistical analysis.

After a preliminary analysis step, five of the samples were deemed clear outliers, so pellet preparation and spectral acquisition was repeated for these samples, to ensure any atypical observation is not due to technical reasons (but, rather, due to actual biological differences). Furthermore, an “average” pellet was prepared for each sampling/tank combination (to represent a virtual “average fish” for each tank), by sample pooling, to improve estimation of the multivariate centroid for each group. The total number of spectra used in the analysis was therefore 383 (each of them obtained, as stated above, by averaging 25 IR spectra).

Attribution of the different IR absorptions to classes of biomolecules was performed using a plot of the correlation between the different spectral features (Fig. S1) and following the information present in Table 2, which was compiled from different sources (Çakmak, Togan & Severcan, 2006; Çakmak et al., 2003; Ceylan, Tanrikul & Özgener, 2014; Palaniappan, Nishanth & Renju, 2010; Palaniappan & Vijayasundaram, 2008; Palaniappan & Vijayasundaram, 2009; Sánchez-Alonso, Carmona & Careche, 2012).

**Table 2:** Main spectral features and associated biomolecules. Table enumerating the spectral features detected in the 400–1800 cm^−1^ range, associated functional groups and main associated components. Other components which are thought to absorb in the same spectral range are also listed.

Peak #	Wavenumber (cm^−1^)	Associated functional group vibration modes	Main components	Other components
1	1740–1750	C = O stretching of esters and aldehydes	triglycerides, cholesterol esters	aldehydes, esters
2	1710	C = O stretching of ketones and carboxylic acids	fatty acids	ketones, carboxylic acids
3	1650	C = O stretching of amides (amide I peak); alkenyl C = C stretching	proteins	unsaturated fatty acids
4	1570–1610	conjugated C = C stretching	unsaturated fatty acids/lipids	aromatics
5	1540	C–N stretching and N–H bending of amides (amide II peak)	proteins	aromatics
6	1460	methylene C–H bending	lipids	proteins, aromatics
7	1455	methyl C–H assymetric bending	lipids	proteins
8	1395–1415	COO^−^ symmetric stretching	fatty acids, amino acids	other carboxylates
9	1300–1310	methyne and olefinic C–H bending	unsaturated fatty acids/lipids	alcohols, aromatic amino acids, organic phosphates, carboxylates
10	1240	${\mathrm{PO}}_{2}^{-}$ assymetric stretching	nucleic acids	phospholipids
11	1150–1155	CO–O–C assymetric stretching of glycogen and nucleic acids	carbohydrates, nucleic acids	aromatics, phospholipids, cholesterol esters
12	1100	C–O stretching of secondary alcohols	carbohydrates, glycerol	aromatics
13	1080	C–O stretching of glycogen; ${\mathrm{PO}}_{2}^{-}$ symmetric stretching	carbohydrates, nucleic acids	phospholipids, aromatics
14	1045	C–O stretching of oligo/polysaccharides	carbohydrates	aromatics
15	1025	inorganic phosphate; C–C skeletal vibrations	side chains of aromatic AA	other aromatics (e.g., polyphenols), phosphate
16	930^a^	C–N^+^–C stretch of nucleic acids	nucleic acids	aromatics, phosphatidylcholine, alcohols, carboxylic acids, amines
17	845–865^a^	carbonate; C–C skeletal vibrations, C–H out-of-plane bend	lipids	aromatics, carbonate
18	760^a^	methylene (CH_2_)_*n*_ rocking; C–C skeletal vibrations	*unknown*	aromatics
19	700–720^a^	methylene (CH_2_)_*n*_ rocking; C–C skeletal vibrations; olefinic C–H; thiols	lipids	glutathione, alcohols, aromatics
20	650^a^	*unknown*	*unknown*	alcohols
21	610^a^	disulfides	*unknown*	glutathione, proteins, alcohols
22	575^a^	*unknown*	*unknown*	*unknown*

**Notes.**

a Changes in this zone of the IR spectrum are difficult to interpret, given the high number of functional group vibration modes present here; besides the ones mentioned in the table, there are also absorptions in this zone related to out-of-plane O–H bending (e.g., from alcohols and carboxylates, which can appear at different wavenumbers, depending on the degree of hydrogen bonding), P–O–C stretching (e.g., from aliphatic phosphates), various modes of methylene rocking and N–H vibration modes, making it challenging to pinpoint observed changes to any particular class of biomolecules.

### Univariate and multivariate data analysis

All data analyses were performed with the R statistical computing software (Ihaka & Gentleman, 1996) and generally taking “fish” as the basic experimental unit, whenever possible. For the zootechnical measurements, differences between means were assessed using one-way ANOVA, taking a significance threshold of *p*-value <0.05 and using Tukey HSD as post-hoc test. For the FT-IR data, spectra were truncated to the 500–1800 cm^−1^ area-of-interest (due to high variability in the 1800–4000 cm^−1^ area), converted from transmittance to absorbance and normalized, by application of a simple SNV (standard normal variate) transform (i.e., mean centering each spectrum, then dividing each spectrum by its standard deviation and finally adding a constant across all spectra to ensure strictly positive values), resulting in a data matrix of 383 spectra × 676 bins per spectrum. Signal-to-noise ratio was estimated based on all the spectra before normalization, as *μ_rep_*/*σ_rep_*, where *μ_rep_* is the mean transmittance value obtained for the different technical replicates of each biological sample (i.e., an estimate of the “signal”), and *σ_rep_* is the sample standard deviation of those technical replicates (i.e., an estimate of the “noise”), across all biological samples and spectral bins (see Fig. S2). After a preliminary analysis showed small (biological) differences at the level of the protein-associated amide I and II bands (1500–1700 cm^−1^), spectra were re-normalized according to the area under those peaks. Univariate statistical analysis of the FT-IR spectra was performed by modeling each spectral bin using linear mixed-effect models (assuming variables “season” and “diet” to display fixed effects and variables “tank” and “fish” to display random effects). Statistical significance was assessed by setting a *p*-value threshold such that the false discovery rate (FDR) was below 1%. Linear mixed-effect models were performed using the lmer function (from package *lmerTest* (Kuznetsova, Brockhoff & Christensen, 2013)). Multiple comparison correction was performed using the qvalue function (from package *qvalue* (Storey, Taylor & Siegmund, 2004)). Multivariate analysis of the FT-IR spectra was performed by calculating an interspectral distance based on Kendall’s tau correlation coefficient (1−*τ*^2^) and applying Sammon mapping, using the sammon function (from package *MASS* (Venables & Ripley, 2002)), to generate an unsupervised low-dimensional embedding of the samples from the dissimilarity matrix. In order to assess potential correlations between zootechnical and FT-IR variables, sPLS (sparse Partial Least Squares) regression was performed using function spls (from package *mixOmics* (Lê Cao, Déjean & González, 2012)).

## Results and Discussion

**Figure 2:**
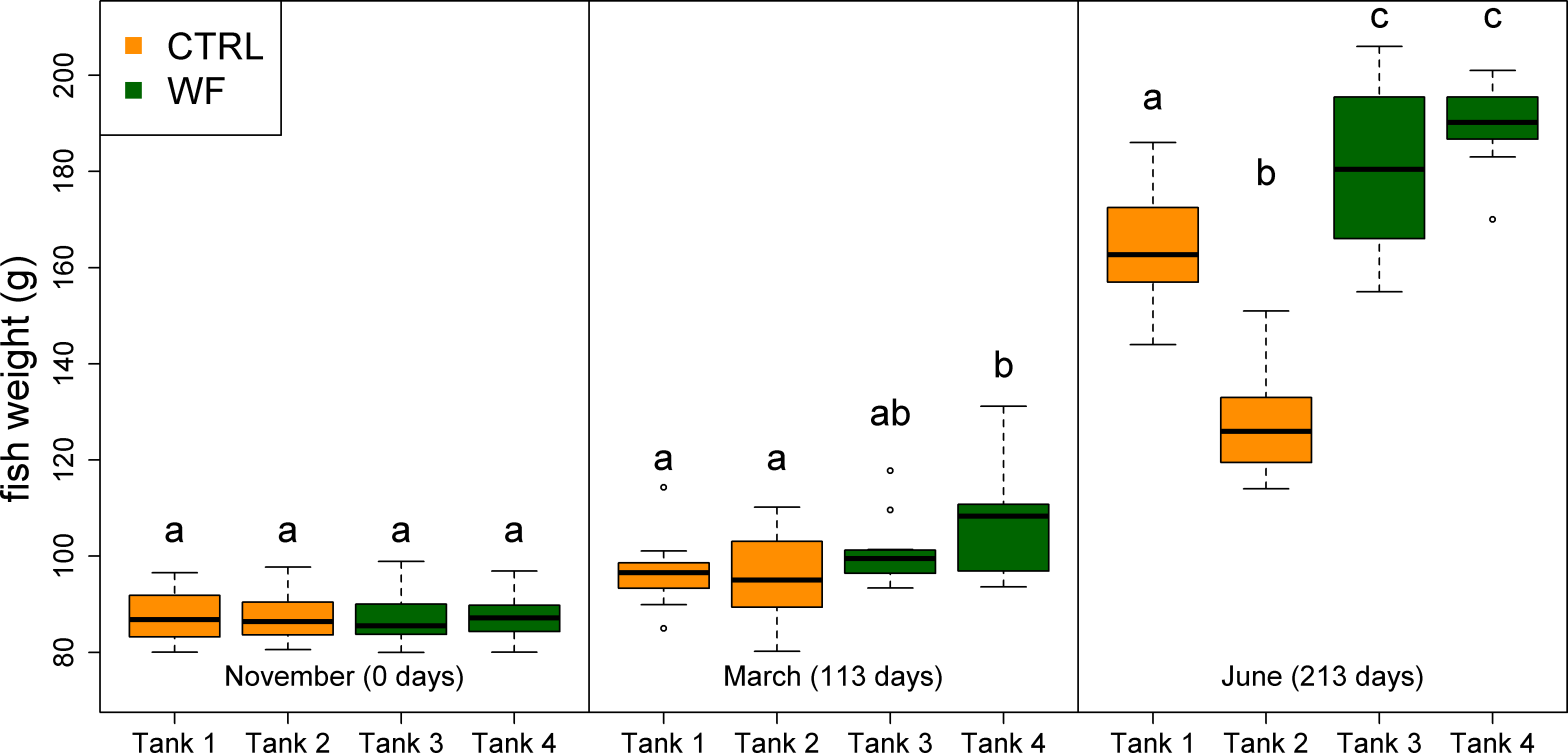
Box plots of the fish weight distributions. Plot showing the distributions of fish weight estimated from individual measurements (*n* = 10 per tank, except for the November data, where *n* = 45), for each separate tank, at each of the sampling points (trial start, 1st sampling and 2nd sampling). Tanks fed with CTRL diet are indicated in orange, while tanks fed with WF diet are indicated in dark green. Differences in means between groups with different letters are statistically significant, as assessed by Tukey HSD test (*p* < 0.05).

**Figure 3:**
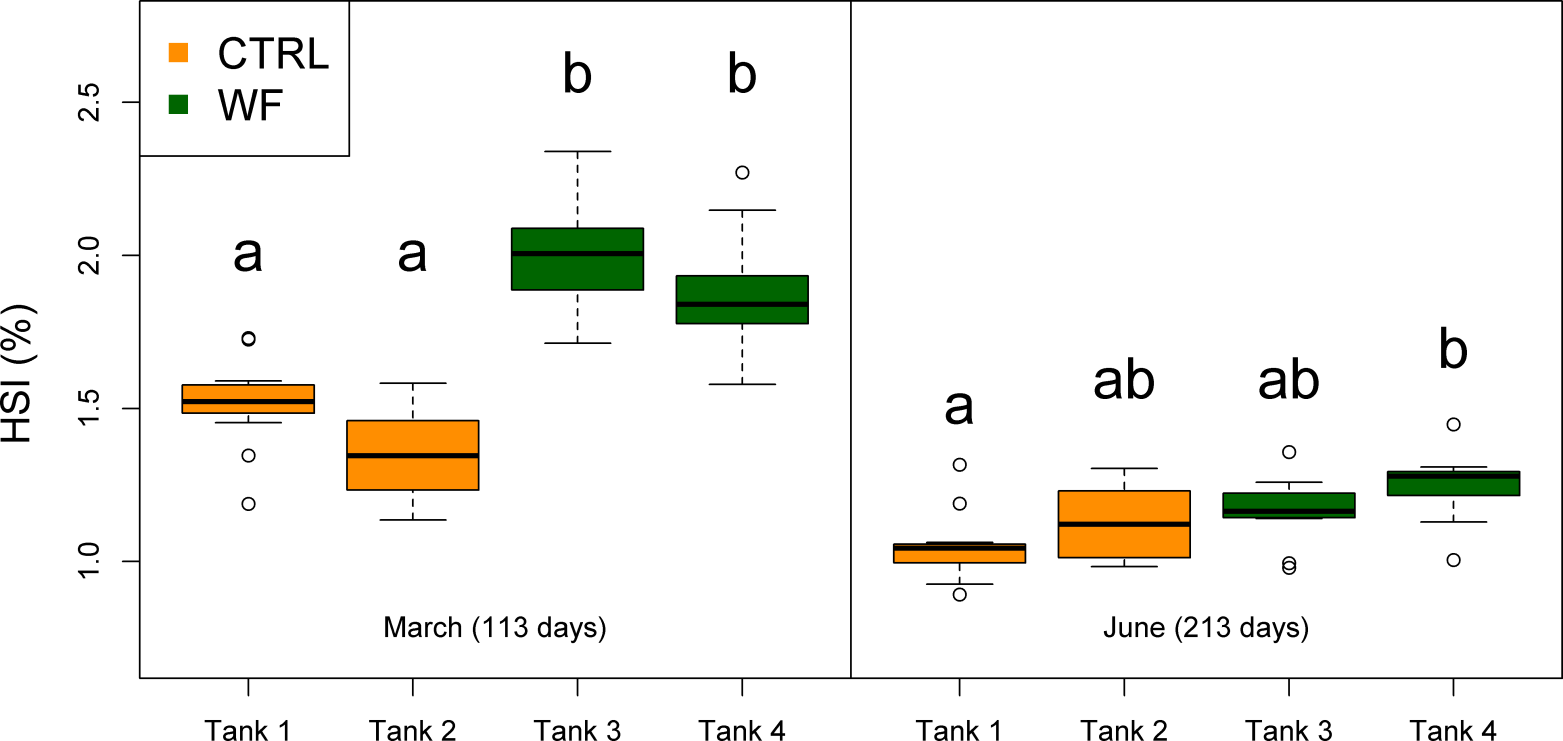
Box plots of the fish hepatosomatic index distributions. Plot showing the distributions of fish hepatosomatic index estimated from individual measurements (*n* = 10 per tank), for each separate tank, at each of the sampling points (1st sampling and 2nd sampling). Tanks fed with CTRL diet are indicated in orange, while tanks fed with WF diet are indicated in dark green. Differences in means between groups with different letters are statistically significant, as assessed by Tukey HSD test (*p* < 0.05).

The results of the fish trial appear to support positive effects of the WF diet on fish performance, with fish fed this diet generally displaying higher relative growth rates (RGR), higher thermal-unit growth coefficients (TGC), higher hepatosomatic indices (HSI) and lower feed conversion ratios (FCR), compared to CTRL-fed fish (see Table 3, Figs. 2, 3 and S3). Though CTRL-fed fish displayed significantly higher feed consumption during the spring period (when expressed as a fraction of body weight), this did not result in any appreciable compensatory growth. It is interesting to note that the TGCs estimated for the winter period are consistently lower than those estimated for the spring period, which suggests that observed differences in terms of growth performance between the two periods should be attributed not only to the temperature differences, but also to the voluntary fasting effect due to low temperatures. No mortalities were reported for any of the tanks, which is a sign that the fish coped with the seasonal challenge to some degree, displaying none of the behavioural symptoms of “winter disease”. Nevertheless, some of the fish (particularly in the CTRL tanks) displayed at least one of the phenotypic traits of “winter disease”: pale and friable liver. No significant differences between tanks were observed in terms of whole body composition (results not shown).

**Table 3:** Bulk performance parameters for the two inter-sampling periods. Table with mean fish weights, feed consumption, daily relative growth rate (RGR), thermal-unit growth coefficients (TGC) and feed conversion ratios (FCR) calculated per tank from bulk measurements, for the two inter-sampling periods. Where present, value spread is expressed as standard error of the mean, calculated assuming *n* = 2. Statistically significant differences in mean between treatments (one-way ANOVA, *p* < 0.05) is indicated with an asterisk.

	Tank 1 (CTRL)	Tank 2 (CTRL)	Tank 3 (WF)	Tank 4 (WF)	CTRL	WF
					**Winter period** **(November 18th to March 7th)**	
Mean initial wet weight (g fish^−1^)	87.0	87.2	87.1	87.2	87.1 ± 0.1	87.2 ± 0.1
Mean final wet weight (g fish^−1^)	97.6	94.4	103.2	109.6	96.0 ± 1.6	106.4 ± 3.2
Tank daily RGR^a^ (%)	0.10	0.07	0.15	0.20	0.09 ± 0.02	0.18 ± 0.03
Tank TGC^b^(10^−3^ g^1/3^ °C^−1^ day^−1^)	0.13	0.09	0.20	0.27	0.11 ± 0.02	0.23 ± 0.04
Mean feed consumption (g fish^−1^)	41.1	37.3	43.9	45.0	39.2 ± 1.2	44.5 ± 0.6
Mean feed consumption (%BW day^−1^)	0.43	0.40	0.44	0.44	0.41 ± 0.02	0.44 ± 0.01
Tank FCR^c^	3.9	5.2	2.8	2.0	4.5 ± 0.7	2.4 ± 0.4
					**Spring period** **(March 7th to June 15th)**	
Mean initial wet weight (g fish^−1^)	97.6	94.4	103.2	109.6	96.0 ± 1.6	106.4 ± 3.2
Mean final wet weight (g fish^−1^)	163.4	128.5	180.9	186.5	146.0 ± 17.5	183.7 ± 2.8
Tank daily RGR^a^ (%)	0.53	0.28	0.58	0.59	0.41 ± 0.13	0.59 ± 0.01
Tank TGC^b^ (10^−3^ g^1/3^ °C^−1^ day^−1^)	0.70	0.40	0.78	0.75	0.55 ± 0.15	0.77 ± 0.02
Mean feed consumption (g fish^−1^)	171.5	136.0	140.7	142.8	153.8 ± 17.8	141.8 ± 1.0
Mean feed consumption (%BW day^−1^)	1.91	1.80	1.43	1.45	1.86 ± 0.06	1.44 ± 0.01^∗^
Tank FCR^c^	3.3	5.0	2.4	2.1	4.2 ± 0.9	2.3 ± 0.2

**Notes.**

a Relative growth rate, calculated as RGR (%) = 100 × (e^(ln(*Wf*)−ln(*Wi*))/(*tf*−*ti*)^−1), where *W_i_* and *W_f_* are the mean initial and final fish wet weights, while *t_i_* and *t_f_* are the initial and final times of the growth period, respectively.

b Thermal-unit growth coefficient, calculated as TGC $(1{0}^{-3}~{\mathrm{g}}^{1/3}~\deg {\mathrm{C}}^{-1}~{\mathrm{day}}^{-1})=1000\times ({W}_{f}^{1/3}-{W}_{i}^{1/3})/\mathrm{DD}$, where *W_i_* and *W_f_* are the mean initial and final fish wet weights, respectively, and DD is the sum of degree.days for the period.

c Feed conversion ratio, calculated as FCR = FC/(*W_f_*−*W_i_*), where *W_i_* and *W_f_* are the mean initial and final fish wet weights, respectively, and FC is the mean feed consumption.

**Figure 4:**
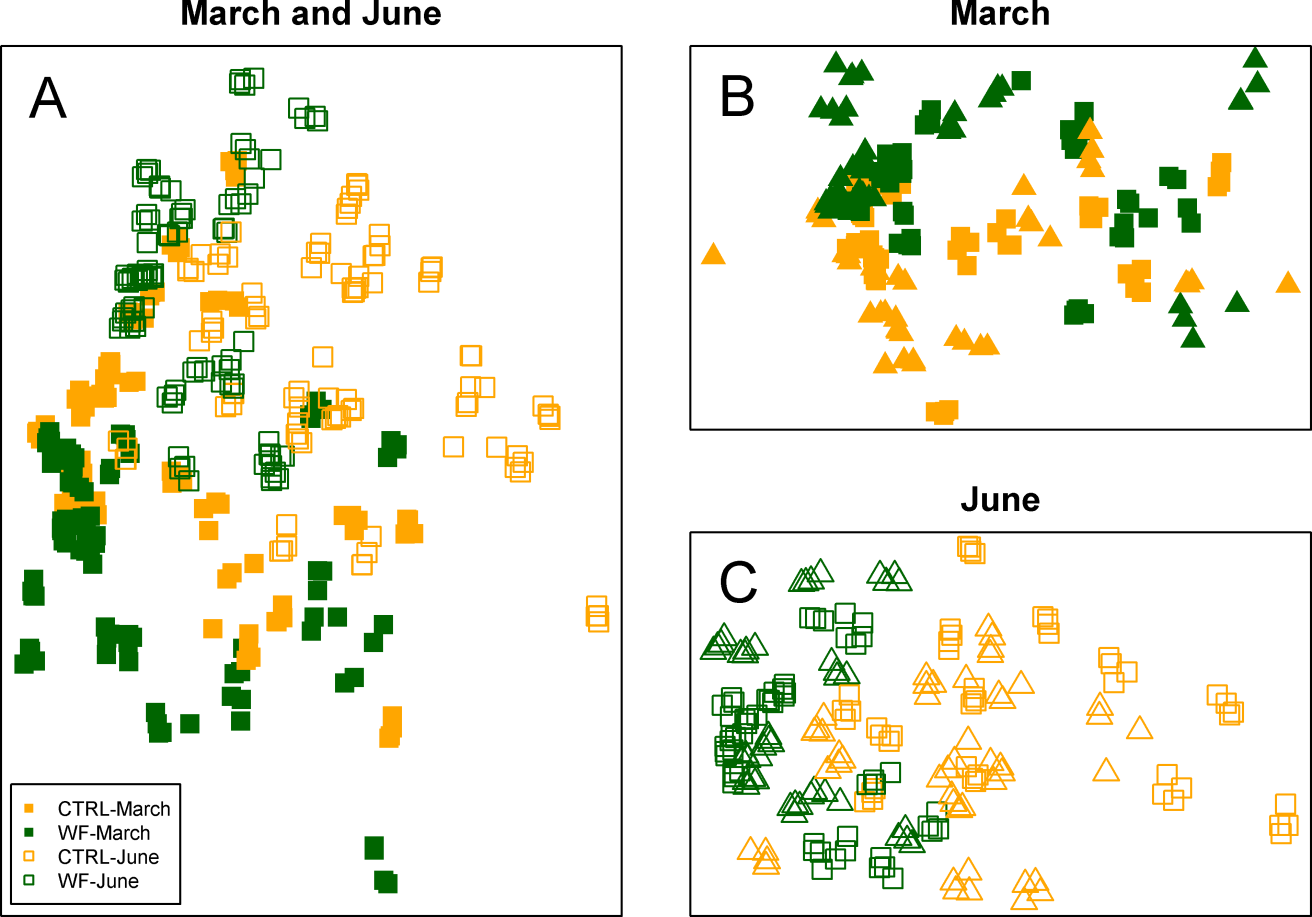
Clustering of the FT-IR spectra. Two-dimensional embeddings of the samples (*n* = 383), obtained by Sammon mapping of the FT-IR dataset using a dissimilarity measure based on Kendall’s correlation, for the two samplings, either together (A) or separately (B and C). The symbols correspond to the sampling times (filled symbol for 1st sampling and empty symbol for 2nd sampling), while the colours correspond to the treatments (orange for CTRL-fed tanks; dark green for WF-fed tanks). For (B) and (C), samples from different tanks are distinguished by the use of distinct symbols.

Multivariate analysis of the FT-IR dataset suggests that the biggest observed differences in terms of hepatic metabolic fingerprint are between samples from the June *vs.* March sampling (Fig. 4A). Unsupervised embedding of the samples from the two samplings in separate also shows a clear effect of the WF diet *vs.* CTRL diet (Figs. 4B and 4C), particularly for the June sampling. Comparatively, no clear tank effect (i.e., when comparing Tank 1 *vs.* Tank 2 and Tank 3 *vs.* Tank 4) on the hepatic metabolic fingerprints can be observed.

**Figure 5:**
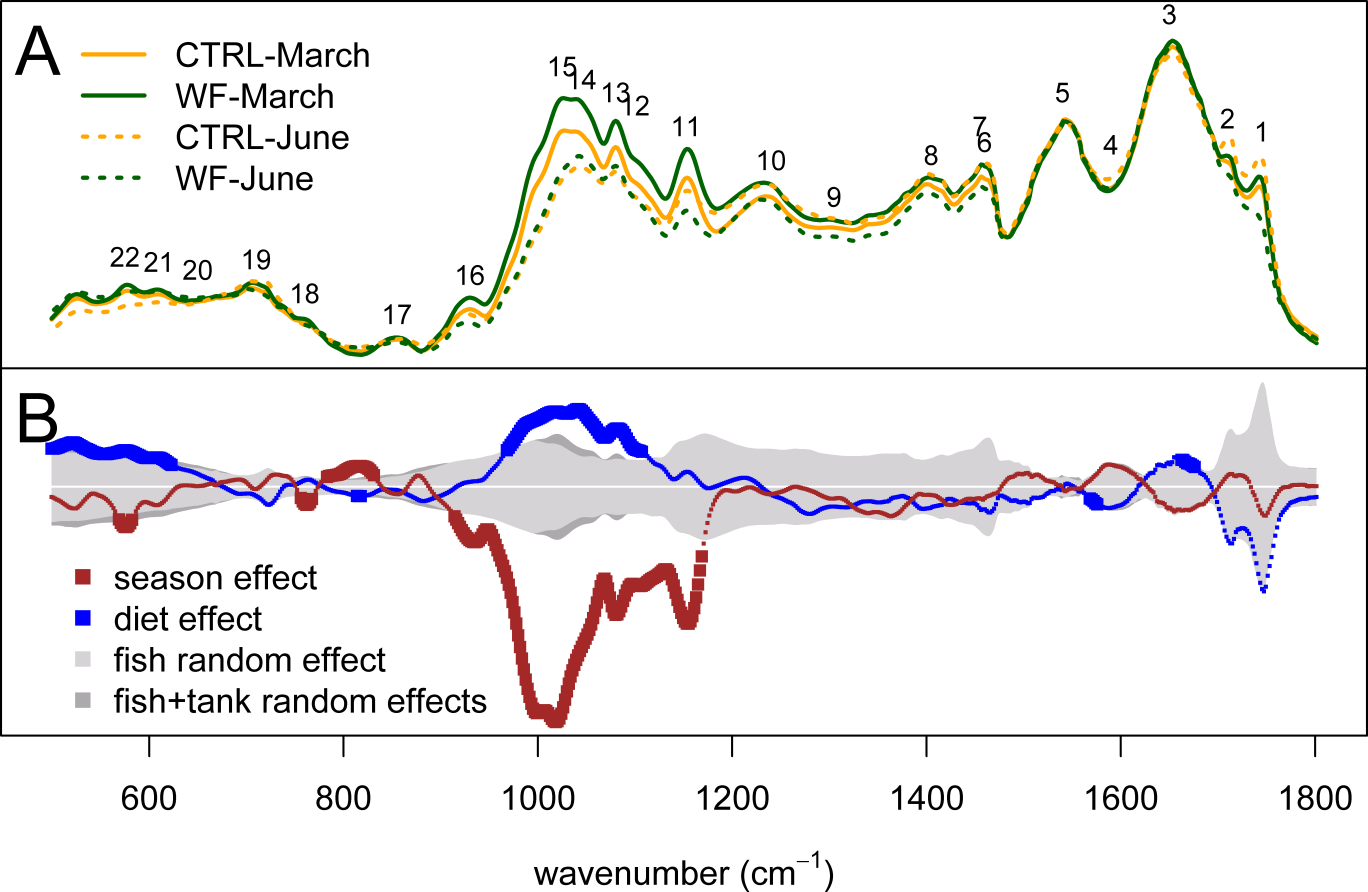
Univariate statistical analysis of the FT-IR spectra. (A) Plot of the average spectrum for each dietary treatment (orange for CTRL and dark green for WF), at each of the two sampling points (full line for the March sampling and dotted line for the June sampling). (B) Plot showing, for each spectral bin, the magnitude of the “season” (dark red) and “diet” (blue) fixed effects, compared to the average magnitude of the “fish” random effect (light gray) or a sum of the average magnitudes of the “fish” and “tank” random effects (dark gray). Effect sizes were estimated from the coefficients of the fitted linear mixed-effects model. The dark red and blue lines are thicker for the spectral bins for which the observed effect was considered statistically significant (FDR < 0.01, *n* = 10 per tank per sampling).

Looking at the actual spectra and the results of univariate analysis (Fig. 5), it becomes clear that the biggest season and diet effects can be explained in terms of peaks 11–16 (i.e., 930–1155 cm^−1^), which correspond to IR absorptions attributable to either carbohydrates and/or nucleic acids. If we assume that these IR absorptions correspond to nucleic acids, we would expect to see the same behaviour for peak 10 (highly characteristic of nucleic acids), which does not occur. As such, these observed differences are more likely to represent changes in hepatic carbohydrate content (e.g., glycogen) than changes in nucleic acid content. The results indicate that season had a strong negative impact on the hepatic glycogen reserves of gilthead seabream and that the dietary treatment generally had the opposite effect: not only are carbohydrate stores higher in WF-fed fish compared to CTRL-fed, at the end of winter, but this increase is still visible at the end of spring, despite observed depletion of carbohydrate stores between the two sampling points, for both diets. This type of hepatic glycogen depletion in gilthead seabream due to thermal shifts and fasting has already been documented (Ibarz et al., 2007b).

Another interesting observation regards peaks mostly associated with lipids (both saturated and unsaturated fatty acids, triglycerides, cholesterol esters and phospholipids), such as peaks 1–2, 4, 6–9 and 19, which display an increasing trend over time (i.e., when comparing the March against the June sampling) for the CTRL diet and the inverse trend (decrease over time) for the WF diet, though not significantly so, according to our criterion (FDR > 0.01). Looking at the results in more detail, we can see that this lack of significance is mostly due to high inter-individual variability, with only some of the CTRL fish displaying this trend of increased hepatic lipid content at the end of spring. This observation was confirmed by looking at the 2840–3030 cm^−1^ spectral range (not shown), where lipid-related IR absorptions related to CH stretching are expected to be present, which also displayed the same general trend. This suggests that some CTRL-fed fish appear to be mobilizing lipid stores (probably from perivisceral adipose tissue) at the end of spring, to face increasing energy demands, while none of the WF-fed fish display this effect (suggesting an improved metabolic status). It should be noted that one of the specific symptoms of the “winter disease” is precisely the progressive hepatic accumulation of lipids resulting in a steatotic-like liver (Ibarz et al., 2010c). Though this could be a sign that WF does indeed provide some protective or mitigating effect of the conditions that lead to the onset of “winter disease” in gilthead seabream, no differences were observed in terms of viscerosomatic index to suggest extensive lipid mobilization by CTRL-fed fish, confirming the observation that most fish (regardless of diet) successfully coped with the seasonal temperature challenge, in this particular trial.

**Figure 6:**
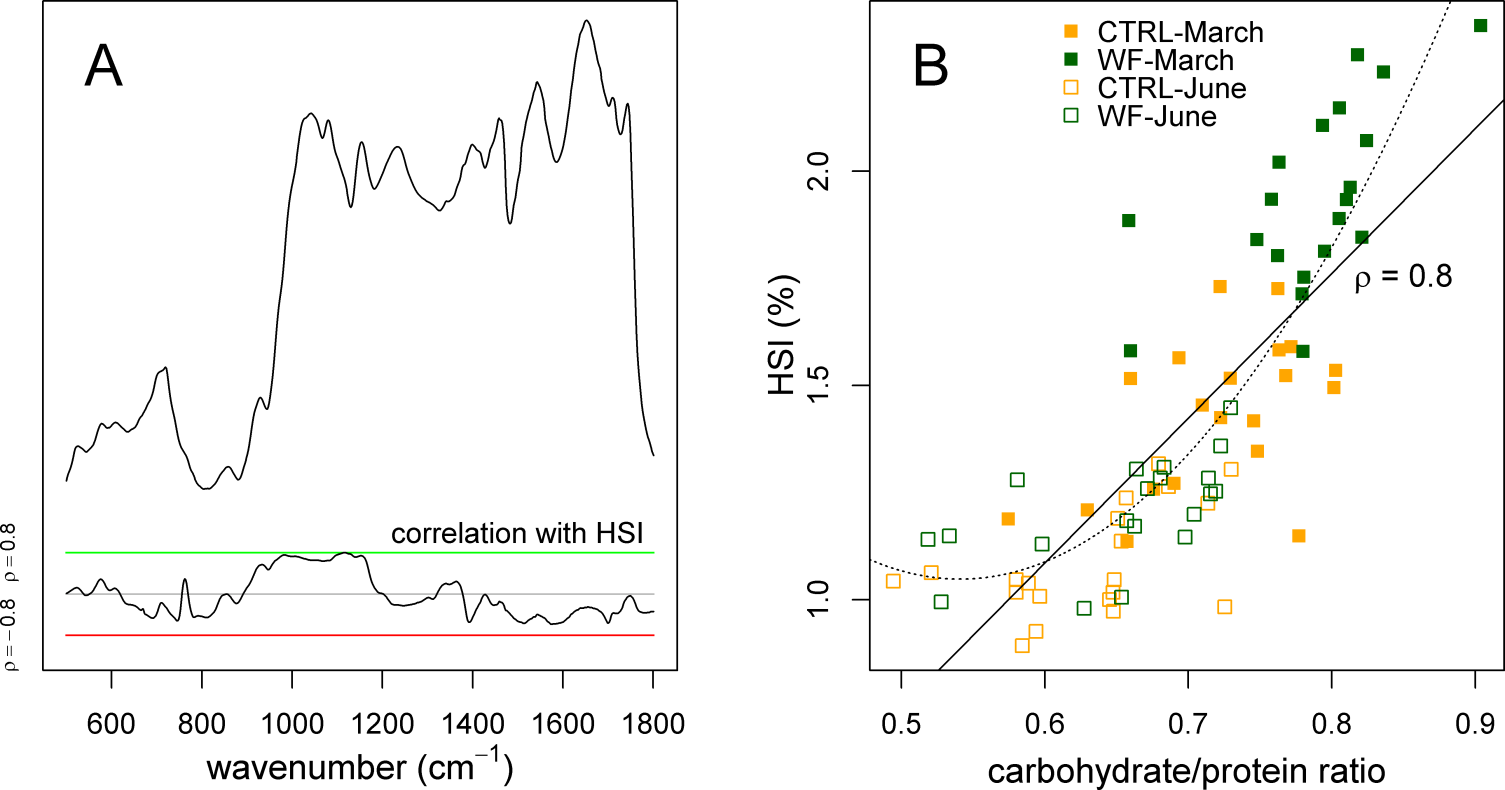
Correlation of hepatosomatic index with FT-IR spectral features. (A) Plot showing a representative spectrum, as well as the correlation of the HSI with each of the spectral bins, across all samples (*n* = 10 per tank per sampling), with a green line denoting *ρ* = 0.8 and a red line denoting *ρ* = −0.8. (B) Scatter plot showing how the HSI relates to the amount of carbohydrates (normalized against protein amount) estimated by FT-IR. Sampling time is identified with a symbol (filled symbol for March sampling, empty symbol for June sampling), while diet is identified with a colour (orange for CTRL, dark green for WF). Lines represent fitted linear (full) and quadratic (dotted) models.

Finally, we also applied sPLS to try to predict the experimental factors and zootechnical parameters (Y-matrix) using the FT-IR dataset (X-matrix). What we observed was that the HSI was the only variable predicted by the FT-IR data with high accuracy. Plotting the correlation of the different FT-IR features against the HSI (Fig. 6A), it becomes clear that there is a strong relation between HSI and the IR absorptions assigned to carbohydrates. This relation can be confirmed by comparing the area under the carbohydrate peaks against the HSI (Fig. 6B), which shows a direct relation between the two (apart from some level of “saturation” for fish with an HSI below 1.25%). This suggests that observed variations in HSI (between diets and between sampling points) can be mostly attributed to changes in hepatic carbohydrate content, again reinforcing the notion that the positive effects observed for WF-fed fish in this trial should be mostly explained in terms of a modulating effect on the hepatic carbohydrate stores.

Interestingly, previous studies on the physiological impact of low temperatures (with fasting) on gilthead seabream (Ibarz et al., 2007a; Ibarz et al., 2005; Ibarz et al., 2007b) suggest that they generally induce an increase in the HSI, which is explained by higher mobilization of lipids (due to higher energy demands caused by thermal shifts). Taken together with the present study, this suggests that increased HSI, by itself, might not be a sufficient predictor of the metabolic state of gilthead seabream, underlining the need to concurrently assess which biomolecules are associated with observed changes in HSI.

## Conclusions

Over the course of this trial, we observed seasonal changes in zootechnical parameters, and in terms of hepatic metabolic fingerprint, which suggest fish were challenged, displaying a progressive loss of hepatic carbohydrate stores. Nevertheless, under experimental conditions, fish still displayed somatic growth over this period and none of the fish displayed all of the symptoms of “winter disease”. Still, some of these, particularly in the CTRL group, displayed possible early signs of the metabolic dysfunction associated to the “winter disease”, namely hepatic accumulation of lipids.

Regarding the WF diet, all obtained information points towards a generally beneficial effect in terms of performance and nutritional/metabolic status, with WF fish consistently displaying higher liver weight and HSI, at both sampling points, mostly due to a difference in the hepatic abundance of carbohydrates. Although the performance parameters (for all tanks) were far from optimal, the WF diet seems to have a mitigating effect regarding the seasonal challenge, not only in terms of impaired growth and carbohydrate depletion, but also in terms of the observed hepatic accumulation of lipids in the later sampling. This effect can be attributed to, among other things, the higher content in fish meal and/or higher protein digestibility, for the WF diet. This suggests that, indeed, the strategy of using a nutritional approach to mitigate the effects of seasonal thermal variations on gilthead seabream metabolism seems feasible and that, in this sense, diet WF is an adequate candidate as a “positive control” diet against which to compare alternate (and possibly more cost-effective) formulations.

Also, we feel confident that the present work reinforces the notion that an assessment of covariates (such as HSI, in this case) on the same set of experimental units can be important and increase the interpretability of observations.

Finally, we have confirmed that the use of FT-IR as a metabolic fingerprinting technology can be useful in such a context to obtain untargeted information on the nutritional and metabolic status of gilthead seabream and other fish species.

## Supplemental Information

10.7717/peerj.527/supp-1 Figure S1 Correlation between spectral features. Plot showing Pearson’s correlation between spectral features (green for “positively correlated”, black for “uncorrelated” and red for “negatively correlated”). For reference, a representative example of a spectra is plotted along both axes. Spectral features are numbered following the same convention as Table 2 and Fig. 5.

10.7717/peerj.527/supp-2 Figure S2 Assessment of technical/instrumental reproducibility. Plot showing estimated signal-to-noise ratio (SNR) as a function of wavenumber. The black line denotes the typical (i.e., median) value across all biological samples, while the grey lines delimit the 95% confidence interval. The vertical axis is in log-scale.

10.7717/peerj.527/supp-3 Figure S3 Zootechnical parameters. Scatter plots showing the relation between individual (*n* = 10 per tank per sampling) fish weight and liver weight (panel A), visceral weight (panel B), HSI (panel C) and VSI (panel D). Symbols correspond to season/sampling time (with filled symbols for 1st sampling and empty symbols for 2nd sampling), while colours correspond to the diet factor (orange for CTRL and dark green for WF).
