# Supplementary figures and images for: Metabolic fingerprinting of gilthead seabream (Sparus aurata) liver to track interactions between dietary factors and seasonal temperature variations
Source: PeerJ. 2014 Aug 26;2:e527. doi: 10.7717/peerj.527 (PMC4157298; doi:10.7717/peerj.527)

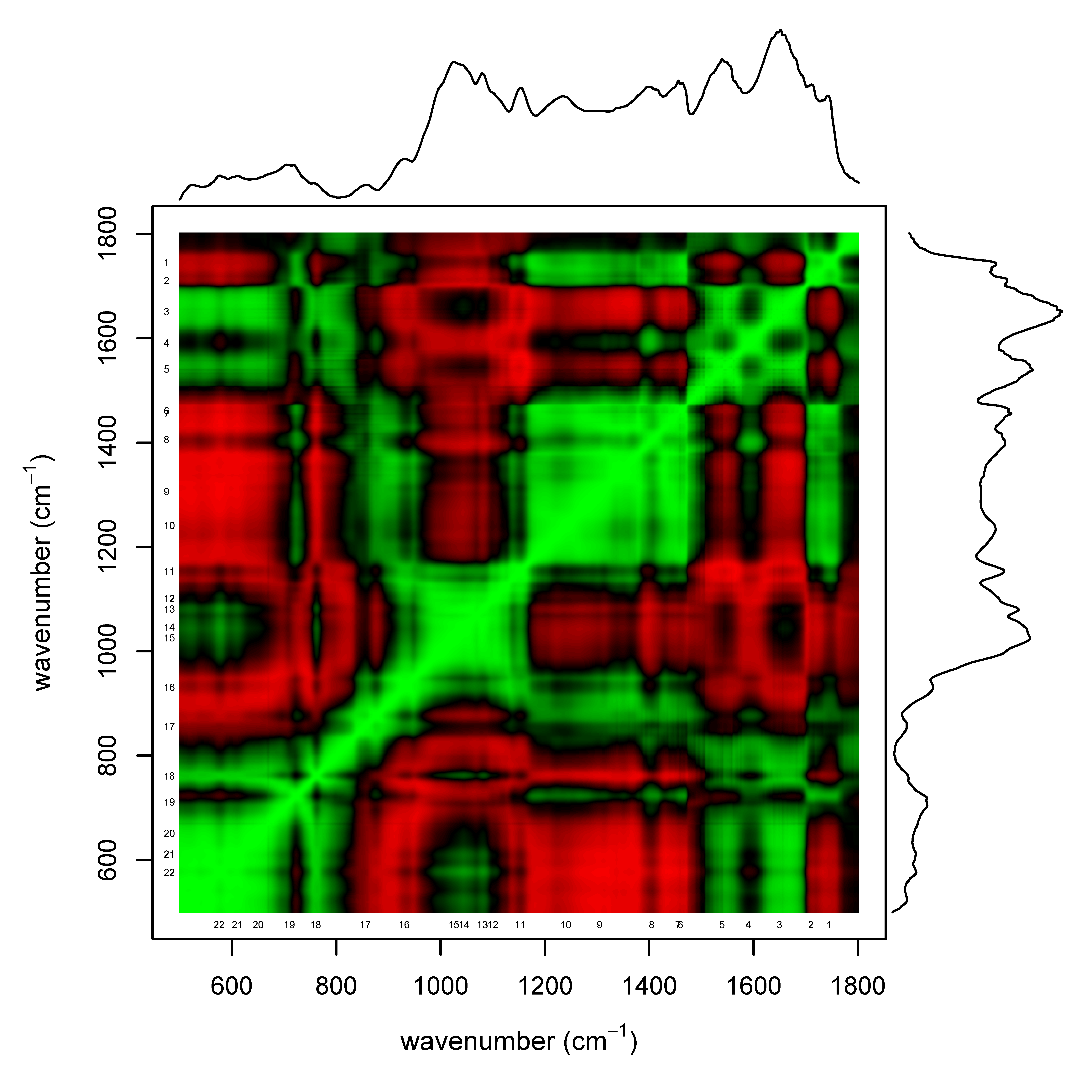

Supplement: Figure S1 — Plot showing Pearson’s correlation between spectral features (green for “positively correlated”, black for “uncorrelated” and red for “negatively correlated”). For reference, a representative example of a spectra is plotted along both axes. Spectral features are numbered following the same convention as Table 2 and Fig. 5. [file peerj-02-527-s001.png]

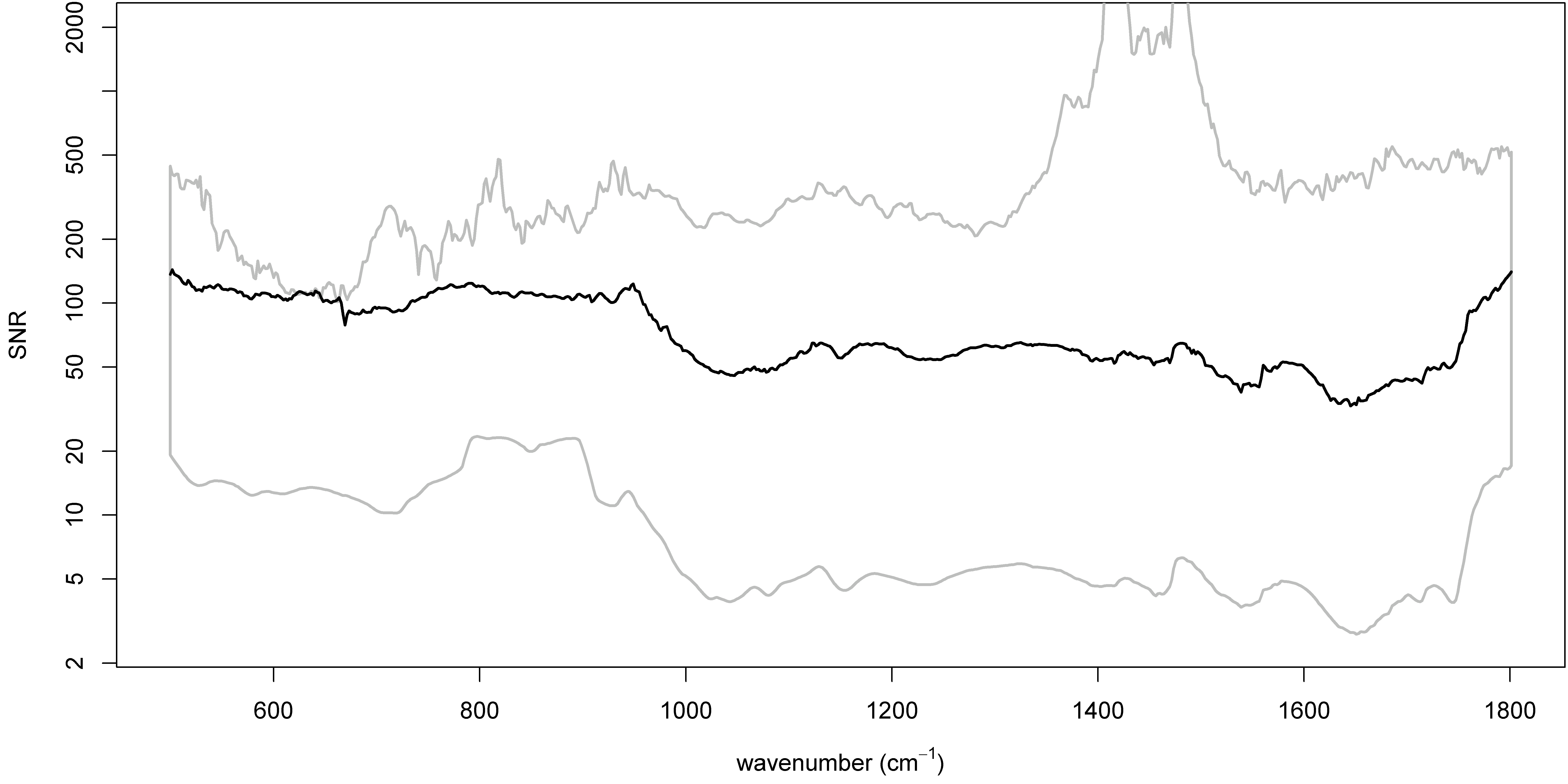

Supplement: Figure S2 — Plot showing estimated signal-to-noise ratio (SNR) as a function of wavenumber. The black line denotes the typical (i.e., median) value across all biological samples, while the grey lines delimit the 95% confidence interval. The vertical axis is in log-scale. [file peerj-02-527-s002.png]
